# Bacterial Cellulose-Based Materials as Dressings for Wound Healing

**DOI:** 10.3390/pharmaceutics15020424

**Published:** 2023-01-27

**Authors:** Manuel Horue, Jhonatan Miguel Silva, Ignacio Rivero Berti, Larissa Reis Brandão, Hernane da Silva Barud, Guillermo R. Castro

**Affiliations:** 1 Laboratorio de Nanobiomateriales, CINDEFI, Departamento de Química, Facultad de Ciencias Exactas, Universidad Nacional de La Plata (UNLP)-CONICET (CCT La Plata), Calle 47 y 115, La Plata B1900, Argentina; 2 Biopolymers and Biomaterials Laboratory—BioPolMat, University of Araraquara—UNIARA, Araraquara 14801-320, SP, Brazil; 3 Max Planck Laboratory for Structural Biology, Chemistry and Molecular Biophysics of Rosario (MPLbioR, UNR-MPIbpC), Partner Laboratory of the Max Planck Institute for Biophysical Chemistry (MPIbpC, MPG), Centro de Estudios Interdisciplinarios (CEI), Universidad Nacional de Rosario, Maipú 1065, Rosario S2000, Argentina; 4 Nanomedicine Research Unit (Nanomed), Center for Natural and Human Sciences (CCNH), Universidade Federal do ABC (UFABC), Santo André 09210-580, SP, Brazil

**Keywords:** wound healing, bacterial cellulose, chronic wounds, cellulose properties, bacterial cellulose structures, bacterial cellulose composites, bacterial cellulose hydrogels

## Abstract

Bacterial cellulose (BC) is produced by several microorganisms as extracellular structures and can be modified by various physicochemical and biological strategies to produce different cellulosic formats. The main advantages of BC for biomedical applications can be summarized thus: easy moldability, purification, and scalability; high biocompatibility; and straightforward tailoring. The presence of a high amount of free hydroxyl residues, linked with water and nanoporous morphology, makes BC polymer an ideal candidate for wound healing. In this frame, acute and chronic wounds, associated with prevalent pathologies, were addressed to find adequate therapeutic strategies. Hence, the main characteristics of different BC structures—such as membranes and films, fibrous and spheroidal, nanocrystals and nanofibers, and different BC blends, as well as recent advances in BC composites with alginate, collagen, chitosan, silk sericin, and some miscellaneous blends—are reported in detail. Moreover, the development of novel antimicrobial BC and drug delivery systems are discussed.

## 1. Introduction

Since the beginning of humanity, wound healing, mainly meaning the healing of the skin, has been recognized as important for health. Papyrus scrolls from ancient Egypt (3200–300 BC) already describe wound treatment procedures using compression for hemostasis, as well as Hippocratic healing techniques, indicating the importance of draining pus from the wound (‘Ubi pus, ibi evacuate’). Furthermore, Galen explains the principles of wound healing by primary and secondary intention. However, much of this knowledge was lost over time and was rediscovered by Brunschwig and von Gersdorff in the modern era. In the late 19th century, the development of antisepsis by Lister and Semmelweis, the detection of pathogenic microorganisms by Koch, and, most notably, the discovery of penicillin by Fleming and sulfonamides by Domagk had an enormous impact on the understanding, therapy, and outcomes of wound healing [1,2]. 

Today, research seeks a deeper understanding of the complex interaction of cells and the distinct influence of different cytokines and growth factors and unfolds the molecular biology of skin wound healing. The goals, even after 5000 years of wound therapy, however, have not changed. The patient still deserves a fast, uncomplicated, and antiseptic wound closure but also desires an aesthetic outcome with unimposing scar formation [1].

Wounds can be defined as the disruption of the normal structure and function of the tissue. In skin wounds, there is disruption of the skin as a result of physical, thermal, or chemical damage, or due to the presence of some underlying medical or pathological conditions [3,4,5,6]. Different wound types can be described by considering several attributes, such as the type of injury, the degree of injury, the duration of the wound, blood flow, infection, inflammation, and other parameters. Depending on the healing time of the wound, they can be classified as acute or chronic [3,4,5,6].

Chronic wounds, or complex wounds, are those that do not progress to the normal healing process, take more than 12 weeks to be healed, and may reappear frequently. Such wounds do not progress to the healing process because they are disturbed during the healing stagesby physiological conditions, such as bacterial infections and associated pathologies [7].

Some factors that complicate the common course of wounds include lack of primary care or poor care, bacterial infections, diabetes, and vascular problems. Chronic wounds include pressure ulcers, diabetic feet, venous ulcers, and ulcers aggravated by obesity; some of them need to be initiated by trauma, like acute wounds. However, the associated conditions hinder the course of the healing process and prolong healing time [2,3,8,9,10,11]. 

Concerning statistical data, according to the document “Wound and lymphoedema management” by the World Health Organization (WHO), based on well-documented data in the USA, about 5–7 million chronic wounds occur per year, corresponding to approximately 2% of the country’s population [8,10]. In 2013, the annual cost of treating chronic wounds was over USD 20 billion, not including additional costs to the population, such as lost productivity and time away from work [12]. Recent data estimated annual spending of more than USD 25 billion in 2018, and almost half of this amount was used for the treatment of pressure ulcers (PUs) [10].

PUs are wounds that occur on the skin and/or underlying tissue and are the result of tissue compression by prominent bones, medical equipment, and/or other objects, associated or not with shear and/or friction forces, in a localized area [9]. Among the groups most vulnerable to developing PUs are the elderly, stroke victims, people with dementia, diabetic patients, and people who are in wheelchairs, are bedridden, or have impaired mobility or sensation. It is estimated that there are more than 7.4 million PUs in the world, excluding data from developing countries. In the United States, about 2.5 million people develop PUs, leading to an estimated treatment cost of USD 11 billion annually [9].

Chronic wounds often affect patients with diseases such as diabetes and obesity [9]. Worldwide, there are about 422 million people with diabetes, and most of them live in low- and middle-income countries [11,12,13].

According to the American Diabetic Association, diabetes affects 20.8 million children and adults, or 7% of the USA population. Specific ethnic or age groups have a higher incidence of diabetes, which has generated great concern: aged 60 years or older, 20.9%; aged 20 years or older, 9.6%; African Americans, 13.3%; Native Americans, 12.8%; Hispanic Americans, 9.5%, and non-Hispanic whites, 8.7%. Diabetics are often prone to chronic wounds and foot ulcers that are difficult to heal; 15% of diabetics have diabetic foot ulcers, which leads to more than 82,000 amputations annually [12,14,15]. The highest expenditures for treating chronic and acute wounds were for treating surgical wounds followed by diabetic foot ulcers, with a higher trend for costs associated with outpatient wound care compared with inpatient [9,16]. Cardiovascular diseases (mainly heart disease and stroke), diabetes and associated chronic wounds, musculoskeletal disorders, and some cancers (including endometrial, breast, ovarian, prostate, liver, etc.) are some of the illnesses associated with obesity and overweight.

In recent years, there has been an increasing interest in developing devices for wound healing, tissue repair, and tissue engineering. This biomedical emergence is based on several considerations, such as the increase in aging people due to a better quality of life; the discovery of novel pathologies related to elderly people; the increase in worldwide pathologies, such as diabetes and respiratory diseases; complex wound pathologies; the limited clinical efficiency of commercial devices; and the high cost of commercial devices not commonly available in low-income countries [17,18]. Based mainly on these considerations, the global market for wound healing was estimated at USD 19.88 billion in 2020, and the compound annual growth rate (CAGR) was expected to grow at 4.1% from 2021 for the next six years [19]. 

Bacterial cellulose (BC) and its different structures and composites are considered appropriate biomaterials for tissue engineering and regenerative medicine, particularly playing a key role in wound healing with some commercial products available on the market. However, there are more novel strategies to create BC-efficient structures for wound healing being explored. Bacterial cellulose has different properties compared with vegetable cellulose, such as high purity, a large surface area, high wet tensile strength, a nanofibrillar structure, biocompatibility, and interesting properties for biomedical applications [20,21,22].

Currently, BC membranes are widely used as dressing devices, marketed under several brands, such as Bionext^®^, Membracell^®^, and Xcell^®^, as they mimic the extracellular matrix to increase epithelialization. They show rapid epithelialization and tissue regeneration rates in wound-healing treatments, such as diabetic foot wounds, chronic wounds, and burns. The treatments of wounds using BC membranes are more efficient compared with conventional gauze or synthetic materials such as Tegaderm^®^, Cuprophan^®^, or Xeroform™ [23].

BC has healing and tissue regenerative properties [24]. It has high in vivo biocompatibility, the ability to provide an ideal three-dimensional substrate for cell fixation, and a microfibrillar structure that provides flexibility, high water retention capacity, and gas exchange, making it a promising biopolymer for wound healing. BC membranes reduce pain and bacterial infection and allow the transfer of drugs to the injured region due to the physical barrier they form [23].

In the treatment of wounds, BC has been used as a temporary substitute for the skin, where besides covering the area and serving as a barrier against external contamination, the membrane maintains local moisture, absorbs exudates, and does not adhere to the wound [21,22,25]. In this way, BC can also be oriented for use in transdermal drug delivery systems.

Despite the excellent properties shown by BC for wound healing and tissue regeneration, the main limitation of BC is its lack of antimicrobial properties, which limits its biomedical applications [2,26]. To remedy this limitation, antimicrobial biomaterials based on BC are being obtained with antimicrobial agents, using physical or chemical approaches in the nanonetwork structure of BC [27,28].

In the present review, the comparative properties of BC and plant cellulose are analyzed. BC’s main properties and the new potential strategies for the development of different BC structures and micro- and nanocomposite devices are reviewed. Moreover, studies of drug-controlled release based on BC are briefly reviewed.

## 2. BC Synthesis, Properties, and Comparison with Plant Cellulose

**Figure 1:**
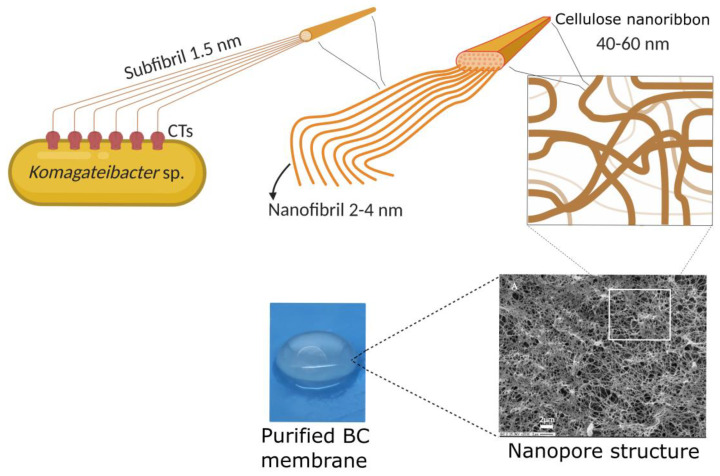
Schematic representation of glucan chain extrusion through CTs and the self-assembly process of the ribbon-like structure formation that entangles to generate the nanopore structure of BC (modified from Cacicedo et al., 2016) [32]. Reprinted from *Mater. Sci. Eng. C, 116*, 111152, Horue, M.; Cacicedo, M.L.; Fernandez, M.A.; Rodenak-Kladniew, B.; Torres Sánchez, R.M.; Castro, G.R. Antimicrobial activities of bacterial cellulose—Silver montmorillonite nanocomposites for wound healing, 2020, with permission from Elsevier [33]. Created by biorender.com, accessed on 7 December 2021.

BC was traditionally known as nata de coco in South-East Asia, where it is consumed as a popular dessert. Nata de coco is produced by aerobic bacteria belonging to the *Komagataeibacter* genus (firstly named Acetobacter, later on reclassified as *Gluconacetobacter* genus) that grow in coconut-water-absorbing nutrients present in the fruit to synthesize a cellulose pellicle on the surface of water media [29]. It is well known that other genera can also produce BC such as Agrobacterium, *Rhizobium*, *Salmonella*, and *Pseudomonas* [23]. Bacterial members of the *Komagataeibacter* genus are, to date, the most efficient BC producers, *Komagataeibacter xylinus* being the most relevant strain [30]. The BC biosynthetic pathway begins with the glucose polymerization catalyzed by cellulose synthase, followed by glucan extrusion through terminal complexes of the bacterial cell (CTs) that are in the cellular membrane. Thus, the excreted chains interact with each other, driven mainly by intramolecular and intermolecular hydrogen bonding, forming subfibrils 1.5 nm wide. The self-assembly fibril process is followed by the subfibril association into nanofibers 2–4 nm wide. Finally, the subfibrils self-assemble into ribbon-like structures that are 40–60 nm wide and 3–8 nm thick (Figure 1) [30,31]. 

**Figure 2:**
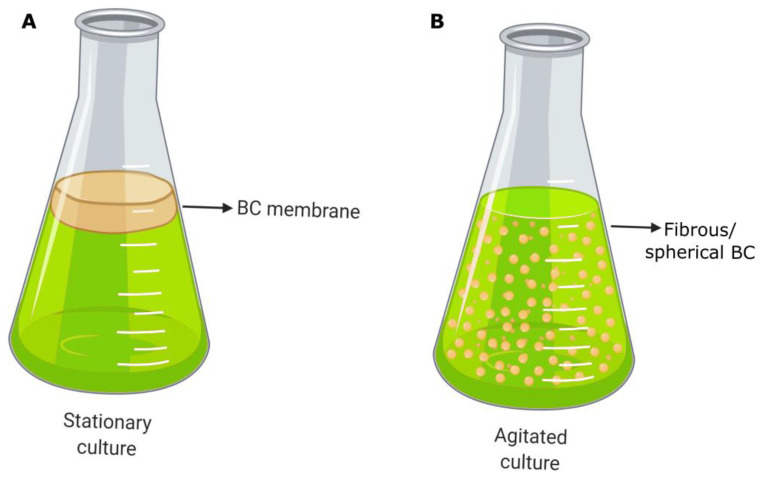
Schematic representation of BC morphologies produced by (**A**) the static method and (**B**) the agitated method (modified from Horue et al., 2018) [31]. Created by biorender.com, accessed on 15 December 2022.

These nanostructures interweave randomly, conforming to a unique nanopore network that is characteristic of this type of cellulose source. Because of this, some authors have named it “bacterial nanocellulose (BNC)” [34,35,36]. If BC microbial producers are cultivated in liquid media by a static method, cellulose is generated in an air/liquid interface as a gelatinous membrane (Figure 2). Naturally, the membrane helps bacterial survival, acting as a protective barrier against adverse environmental conditions (e.g., UV radiation, periods of dehydration, and redox processes) and other microorganisms, or by enhancing the availability of oxygen because of its proximity to the air phase. 

**Figure 3:**
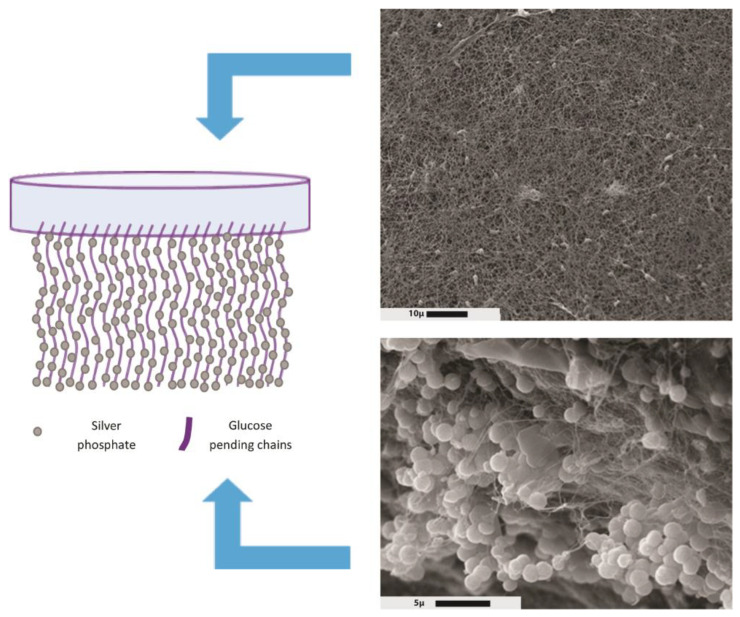
Asymmetric bacterial-cellulose-containing silver phosphate microparticles. Right images: scanning electron microscopy of bacterial cellulose surfaces. Top and bottom: upper and lower membrane sides, respectively. Reprinted from *Colloids Interface Sci. Commun, 26,* Bayón, B.; Cacicedo, M.L.; Álvarez, V.A.; Castro, G.R., Self-Assembly Stereo-Specific Synthesis of Silver Phosphate Microparticles on Bacterial Cellulose Membrane Surface For Antimicrobial Applications, 7–13, 2018, with permission from Elsevier [37].

An interesting characteristic of BC is the asymmetry of the membrane: the BC surface in contact with air is flat, while the membrane surface in contact with the liquid media shows pending chains that can easily be derivatized (Figure 3).

The BC membrane synthesis by a self-assembly process follows well-organized patterns involving the formation of highly crystalline cellulose [31,32]. BC possesses a high crystallinity (more than 70%) with crystals of 5–6 nm, while the remaining percentage corresponds to a less organized phase known as amorphous cellulose. Furthermore, the polymorphism found in BC corresponds to a metastable allomorphism *Iα* exhibiting a high percentage of its crystalline form that is chemically more reactive than allomorphism *Iβ* [38]. From these intrinsic features, remarkable properties arise, such as mechanical strength, thermal resistance, a nanoporous structure, hydrophilicity, high water-holding capacity, biocompatibility, and biodegradability [23,32]. Additionally, from a biomedical point of view, BC has relevant properties for wound healing, such as the stimulation of autolytic debridement of scars, pain reduction, the induction of hyaluronic acid for the acceleration of epithelialization and tissue formation, the absorption of exudates, as well as keeping the wound moisturized and decreasing thrombogenicity [39,40,41].

Cellulose production from woody and nonwoody plants is in the order of 1 × 10^12^ tons per year, making the *Plantae* kingdom the main cellulose source [42,43]. Plant cell walls are constituted by an interspersed matrix of cellulose with other components, such as hemicellulose, lignin, and pectin. A suitable extraction must be performed to remove these impurities and isolate the pure biopolymer. The extraction can involve different kinds of processes, such as mechanical disaggregation, chemical treatment, biological pretreatment, or a combination of them [42,44]. Hence, the whole procedure is energy-intensive, requiring a strong acid/base, and leaking many hazardous wastes or unwanted by-products. This is one of the major drawbacks associated with cellulose obtained from plants, namely, it does not help reduce the carbon footprint and can cause serious environmental issues [44]. BC, however, is a highly pure cellulose source, and purification methods only need to remove bacteria and the rest of the culture media. Procedures only employ alkalis or even NaOCl, leading to softer and more eco-friendly isolation methods [32]. Moreover, although there are no differences in molecular structure between BC and plant cellulose (PC) because both are polymeric chains made up of β-D-glucopyranose units joined by β(1-4) glycosidic linkages, their properties vary considerably. This different behavior is due to various factors: the association of glucan chains differs in plant cellulose (PC), *Iβ* allomorphism being the most abundant crystalline form [38]; the crystallinity index varies according to vegetable source but rarely exceeds 60% [45]; the polymerization degree of the cellulose chains in PC varies between 10,000 and 15,000 glucose units, while in BC it rises to 20,000 glucose monomers [32,38,46]; and the physicochemical treatments applied in the isolation of PC lead to a processed material, while after its purification BC still conserves the reticulated fibrous architecture [47,48].

Consequently, BC presents a superior performance in several aspects: (i) Better mechanical properties; BC tensile strength is in the range of 200–2000 MPa and its Young’s modulus is up to 15–138 GPa (a single BC filament can have a Young’s modulus comparable to Kevlar^®^ and steel) [23,49]. (ii) A higher thermal resistance allows the heating of the biopolymer to over 100 °C and sterilizing it through a conventional autoclaving process (an important requirement desired in the biomedical field). (iii) The exclusive nanoporous structure of BC and the hydrophilic nature of its polymeric chains provide a highly hydrated matrix; for this reason, it is considered a natural hydrogel capable of containing 100–200 times its weight of water [50,51]. Additionally, this type of cellulose has a larger surface area (>150 m^2^/g) and greater porosity (>80%) than PC (surface area of <10 m^2^/g, and porosity of <75%) [52]. The large surface area, in combination with the increased reactivity of allomorphism *Iα*, results in a more suitable material for chemical derivatization [37,53]. (iv) The simple and soft purification methods generate a highly pure material free of hazardous compounds and with the absence of proteins or bacterial remains, thus very low endotoxin units and non-cytotoxicity [23].

Moreover, several biocompatibility assays employing diverse cell lines showed good association, spreading, and proliferation into the BC matrix [54,55,56]. The implantation of BC into living organisms, using diverse in vivo models, demonstrated a low inflammatory response, the absence of a foreign body reaction, blood compatibility, and the absence of hemolysis [54,57,58]. Thus, BC presents better biocompatibility than its counterpart PC. For this reason, BC is considered an excellent material for biomedical applications.

The properties mentioned above may vary to a lesser or greater degree depending on the BC microorganism producer, type of culture method or reactor, culture media, and fermentation conditions [59,60]. Thus, the biotechnology process of BC production is customizable on many levels, and it is possible to obtain biopolymers with different features, morphologies, and shapes. Therefore, a broad range of BC devices with distinct functions for many applications can be fabricated.

## 3. BC Structures

### 3.1. BC Membranes and Films

The most common BC format is the membrane or film produced by microorganisms and cultivated using a static method during the fermentation process, which is the primary option selected on a laboratory scale. As previously mentioned, the membranes are generated on the surface of liquid media due to the accumulation of carbon dioxide by bacterial metabolism that allows them to float (Figure 2A). This production strategy consists in filling the reactor with fresh nutrient media and with bacterial inoculum. Later, the incubation proceeds for a variable period, usually between 1 and 14 days, until membranes with the desired thickness are developed. Finally, the membranes are harvested and subjected to the purification process. In terms of reactors, they are not very sophisticated, and if a larger scale is considered, those most often employed are horizontal lift reactors, aerosol bioreactors, and rotary disc reactors [52,61]. The geometry of BC is determined by the reactor or tank where the culture is carried out. Thus, the modification or change applied to the container is simply reproduced in the morphology of the BC synthesized, and membranes with the desired shapes and sizes can be fabricated easily. This has biomedical relevance because personalized treatments can be applied to individual patients. On the other hand, the low rate of production and its high cost are the main disadvantages of the static culture method [62].

BC membranes have applications in diverse fields, such as biomedicine, pharmaceutics, the food industry, cosmetics, and electronics. However, one of the most relevant areas is biomedicine due to its high added value. BC has huge potential in the biomedical and pharmaceutical areas since it is possible to fabricate advanced devices for wound healing, tissue engineering, drug delivery, and biosensing [23,30]. Specifically, BC membranes can be developed to provide high-performance wound dressing platforms for the treatment of diverse skin injuries or diseases. Hence, some features that benefit bacterial survival in nature help to maintain a suitable wound environment for the healing process. The highly hydrated state of the BC membrane, due to its great water-holding capacity (WHC), provides the appropriate moisture that accelerates the epithelialization rate. Moreover, this humidity diminishes the pain when the dressing is removed or changed because it can reduce scars in the wounded area. Exudates, commonly released by the wound, have negative effects on the injured tissue in recovery, and BC membranes have the absorptive capacity to remove them, resulting in a better healing process. The high porosity of membranes allows for excellent permeability to gasses between the atmosphere and wound, which is necessary for proper re-epithelization.

Due to their mechanical stability and flexibility, they adapt to different parts of the body [54,63]. Despite these remarkable features, BC lacks the necessary activities to produce multifunctional devices with optimal efficiency, and membranes can be improved by generating new systems with better or new properties. In this sense, it is possible to load many molecules with biological activity into membranes due to their nanoporous structure and high WHC [23,30,46,64]. One of the preferred activities conferred to them is antimicrobial activity, which can prevent the colonization of pathogens over the wound or even eradicate it from an already infected wound. Usually, this strategy is accompanied by the design of a hybrid drug delivery–wound dressing system because antibiotics, metals, and nanoparticles are the main compounds loaded into membranes and released over the wound. The modification of the BC matrix through the inclusion of other polymers or inorganic materials is commonly conducted. It has several purposes, including the enhancement of mechanical properties; alteration of some physical parameters such as WHC, water retention rate (WRR), and water vapor transmission rate (WVTR); or even conferring antimicrobial activity [30,32,46,65,66,67]. Moreover, the addition of these materials to the cellulose matrix can alter the normal holding capacity and kinetic release of bioactive molecules due to specific interactions between them, giving the possibility of generating hybrid systems for the controlled release of drugs [23,30,32,46,65].

Modification of BC membranes is not only restricted to producing novel wound dressings and drug delivery systems, although some of them are oriented to enhancing the contact between different cell lines and glucan chains to fabricate scaffolds for tissue engineering. On the one hand, there are strategies directed to changing the porosity and pore size of native membranes to ensure suitable spreading and proliferation of each cell line. To achieve this purpose, useful compounds, known as pore generators, are added at the beginning of the fermentation process. During membrane development, a pore generator interferes in the normal self-assembly process of cellulose chains, altering the nanoporous network. Finally, during the purification procedure, the pore generator is removed, and the resulting membrane possesses a highly porous structure or a bigger one. On the other hand, there are strategies to improve the specific association between cells and the BC matrix that employ chemical derivatization over the glucan backbone or use highly biocompatible molecules to modify membranes [54,63,65]. The great diversity in chemical functionalization, associated with PC, is transferable to BC. A wide variety of reactions can be applied over carbon atom numbers 2, 3, and 6 in glucose-monomer-producing families of BC derivatives. In terms of biomedicine and pharmacology, the most relevant derivatives are cellulose acetate, cellulose sulfate, carboxymethyl cellulose, cellulose nitrate, methyl cellulose, and ethyl cellulose [68].

In conclusion, BC membrane production is a multistage biotechnology process in which each step, from the design of culture media composition to the last modification related to devising production, is customizable. Due to this versatility, it is possible to obtain an entire range of BC composites with special activities and properties directed to their final application.

### 3.2. Fibrous/Spherical BC

If BC production is carried out in agitated or stirred conditions, instead of the static method, the resulting BC has different small shapes that vary from dispersed amorphous fibers to spherical structures of variable size (Figure 2B). The generation of each variant and its features depends mainly on the strain of the BC producer and stirring conditions (specifically on rotation speed). Agitated cultures emerge as a necessity for improving the low rates in BC production associated with static cultures and to scale up the process, achieving a feasible industrial production. In this case, more bacteria can attract oxygen from the air, leading to faster cellulose synthesis. However, this method can develop nonproducing strains during the stirring, causing a yield detriment [59,62].

The physicochemical properties of this BC type differ from those of the membranes mentioned previously. Fibrous/spherical BC presents lower crystallinity, polymerization, and Iα allomorphism associated with a higher Iß content. Furthermore, this cellulose source shows a more open nanoporous network with larger pores, and the direct consequence observed is a higher WHC and surface area [59]. These differences impart a high efficiency in absorbing and carrying structures of varied nature such as compounds, metals, enzymes, nucleic acids, and cells. Thus, they have applications in drug delivery, heavy metal adsorption, cell suspension culture, enzyme immobilization, and tissue engineering [52].

### 3.3. Regenerated Bacterial Cellulose (RBC)

This novel strategy consists of the partial dissolution of BC employing appropriate solvents such as ionic liquids; *N*-methylmorpholine-N-oxide (NMMO) monohydrate; N, N-dimethylacetamide (DMAc)/lithium chloride (LiCl); and NaOH/urea. After that, the BC solution is converted into different structures, such as films, fibers, sponges, and gels by the casting method or wet spinning technology. Although this regeneration process destroys the nanoporous network of BC, it is possible to obtain materials with more structural diversity [69,70]. In this sense, in the bibliography, there are reports of films with an altered nanoscopic architecture that show different cellulose nanoribbon associations, porosity, and pore size. There are even reports of fibers with an ultrafine well-ordered structure that possess improved mechanical properties [70,71,72,73]. The arrangement of cellulosic chains during regeneration makes them adopt the more stable polymorphism cellulose II that is detected in regenerated bacterial cellulose (RBC) derivatives, and they present lower crystallinity and smaller crystallite sizes in comparison with native BC. In addition to structural diversity, another advantage of this strategy is derived from the deeper integration between a reinforcing agent or added compound and BC in its dissolved state. Hence, the properties and functionalities altered by the regeneration method are greater than those obtained by conventional modification strategies. On the other hand, this strategy allows the use of some materials that present incompatibilities with traditional procedures [48,72].

If RBC is compared with its analog, regenerated plant cellulose (RPC), RBC presents better performance related to structural and physiological features [48]. The presented technique allows the production of a new generation of BC devices, increasing the applicability of this biopolymer in the biomedical field.

### 3.4. Cellulose Nanocrystals (CNCs) and Cellulose Nanofibers (CNFs)

Cellulose nanomaterials can be obtained by selective acid or partial enzymatic hydrolysis of amorphous regions, preserving crystalline domains. Specifically, a strong BC hydrolysis treatment leads to highly pure crystals in CNCs, while some amorphous regions remain in CNFs by softer hydrolysis. This feature is related to the dimensions of each nanomaterial: CNCs are rod-like objects with a short diameter (10–30 nm) and a more variable length (i.e., hundreds of nanometers); CNFs are nanosized fibers with a diameter less than 100 nm, while the length is variable too, with a minimum value of 500 nm [74]. A parameter related to the size that allows these nanomaterials to be compared is the aspect ratio (L/D), which is defined as the relation between length and width. In this context, CNCs have an aspect ratio between 5 and 50; they are shorter and more rigid. CNFs have an aspect ratio greater than 50, and they are longer and more flexible [75]. Both structures can be produced from PC and BC; nevertheless, the way they form is substantially different. In PC, CNCs and CNFs can be obtained by a top-down approach where hydrolysis is carried out over cellulose suspensions during the biopolymer isolation from raw materials. Moreover, these nanomaterials are generated during the self-assembly of cellulose nanoribbons by the bottom-up method in BC [68,76]. Some features can vary between nanomaterials from the two main sources: CNCs isolated from BC showed a higher aspect ratio and crystallinity than CNCs isolated from PC [77,78]. In terms of properties, the molecular arrangement of cellulose chains within nanoribbons leads to an amphiphilic nature of CNCs and CNFs. Therefore, they can stabilize the interface of oil-in-water emulsions through a Pickering mechanism. These nanoobjects have low gas permeability in the form of dry film, and this gas barrier property would allow the protection of oxygen-sensitive molecules in free water systems [75,79]. Similarly, they have a large surface area of plenty of hydroxyl groups, like native BC, which are derivatized to improve or impart distinct properties and enhance their dispersibility or colloidal stability in aqueous media. The most common chemical modifications are oxidation, etherification, esterification, carbamation, amidation, silylation, and polymer grafting [76,79]. The major potential of these nanostructures lies in their ability to enhance the mechanical properties of other polymeric matrices, acting as reinforcing agents. Thus, it is possible to fabricate a wide range of stronger hybrid hydrogels. However, the CNC and CNF behavior differs. Due to the low aspect ratio of CNCs, they cannot form stable hydrogels themselves, and chemical derivatization or cross-linking must be performed to reach a consistent network. They are more suitable as fillers for other polymeric matrices. On the other hand, the longer size and semicrystalline nature of CNFs allow them to form consistent hydrogels. This tendency, however, leads to a harder blending process related to hybrid composite formation [74]. The fabrication of hybrid hydrogels can employ several kinds of techniques. Among them are blending or homogenization, solution casting, melt blending, freeze–thawing, free radical polymerization, UV/ion-mediated cross-linking, electrospinning, and even 3D printing [74,76].

CNCs and CNFs are multifunctional, biodegradable, and biocompatible materials able to form films, hydrogels, foams, nanosystems, microparticles, and Pickering emulsions. The obtained devices have applications in biomedical and pharmaceutical areas such as wound dressings; drug delivery systems (especially for poorly soluble drugs); nucleic acids, proteins, enzymes, antibodies, and cell immobilizers; and scaffolds for tissue engineering [23,74,75,76,79].

## 4. BC Hybrids and Composites

**Figure 4:**
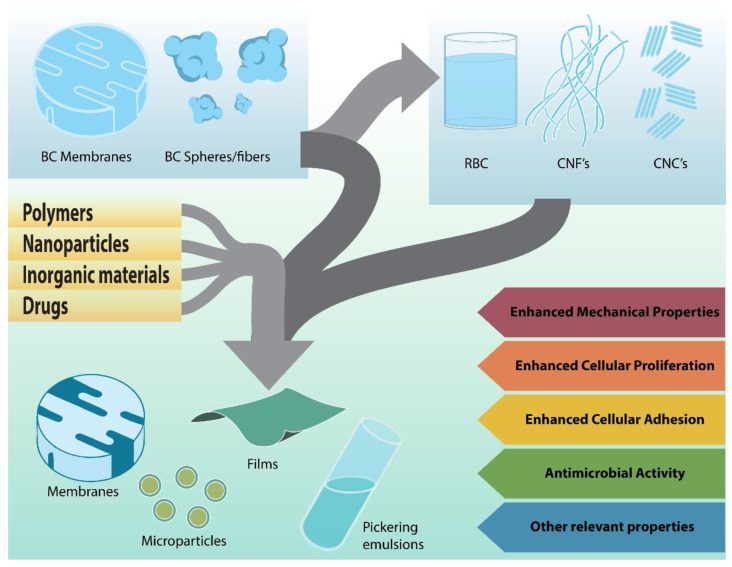
Strategies for the development of bacterial cellulose composites.

A composite material is prepared from two or more materials to obtain combined properties or properties superior to those of the original materials separately (Figure 4).

The main properties that make BC a material with excellent characteristics for wound treatment were reviewed above. BC properties can be improved, their disadvantages can be overcome, and new characteristics can be added to enhance their performance through the synthesis of BC composites. The methodologies to produce BC composites are wide ranging and depend not only on the potential application but also on the partner material. Typically, a myriad of compounds can be added to BC, for example, polysaccharides, proteins, peptides, nanoparticles, tracers, enzymes, antibiotics, and bioactive molecules, among others. Some BC composites developed for several biomedical purposes were recently reviewed [80,81,82].

### 4.1. BC–Alginate Composites

Alginate is produced by many natural sources, including bacteria, but for industrial applications it is obtained from algae (i.e., *Phaeophyceae*, brown seaweeds). Alginate is composed of α-L-guluronic and ß-D-mannuronic acids linked through (1,4) glycosidic bonds in different proportions according to the source. In the presence of multivalent cations (e.g., zinc, calcium, etc.), it can form gels, with a structure usually described as an “egg-box” [83]. Several structures of alginate gels, from fibers, sponges, and microspheres to nanocapsules, can be made by biophysical modifications. Alginate is a biopolymer considered GRAS (Generally Recognized as Safe) by the FDA (Food and Drug Administration, USA), and be used in diverse biomedical applications, such as wound healing, tissue engineering, and drug delivery devices. Among the advantages, alginates make mucoadhesive gels due to their low surface tension (i.e., 31.5 mN/m), which is below that of mucin. However, the loss of water the alginate hydrogels experience when exposed to bivalent cations can destroy interchain H-bonds and irreversibly crack the gel [84,85]. For this reason, alginate is commonly associated with many hydroxylated polymers to make hybrid structures such as coacervates and blends. Many hybrid structures of alginate composites used to make micro- and nanocomposites, bioglass, and ceramics were recently reviewed. Several alginate composites with polymers such as carbon nanotubes, carrageenans, chitin, chitosan, methacrylate, polyethylene glycol (PEG), poly (L-lactide-co-glycolide) (PLGA), pullulan, polyvinyl alcohol (PVA), and starches were reported [86,87]. Similarly, alginate composites containing proteins such as bovine serum albumin (BSA), collagen, and gelatin coupled with peptides such as RGD or growth factors to stimulate tissue repair and cell growth were described [88].

**Figure 5:**
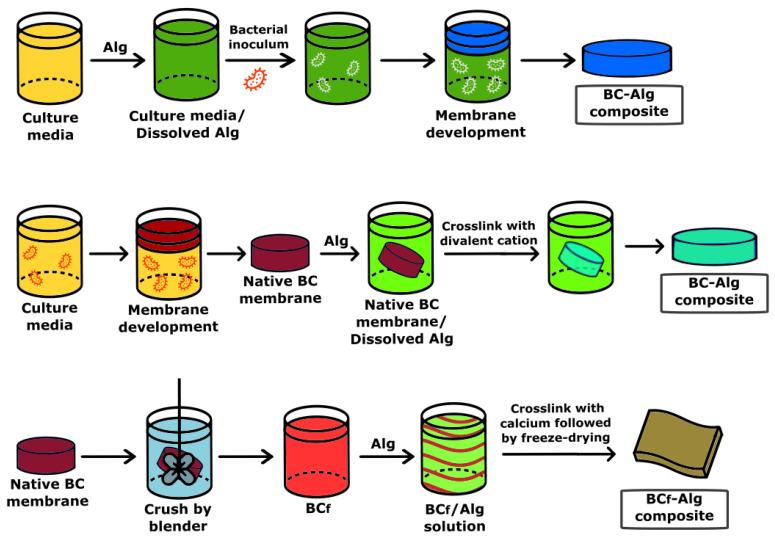
Example of the strategies developed to produce BC–alginate composites.

In addition, antimicrobial BC–alginate (BC-Alg) composites containing biocide or biostatic compounds such as ZnO, copper, silver nanoparticles, lemongrass oil, curcumin, and antibiotics such as ciprofloxacin, mupirocin, natamycin, and silver sulfadiazine, among others, were produced [89,90,91,92,93]. The authors claimed that these BC-Alg composites seem to be appropriate for wound healing and tissue regeneration. Moreover, the experiment results, mostly obtained by in vitro experiments, provided a new opportunity for the development of different strategies to obtain new types of BC-Alg micro- and nanocomposites. Particularly in recent years, the development of BC-Alg composites containing no other molecules or polymers for wound dressing was explored intensively. Three main strategies to produce BC-Alg composites were reported (Figure 5).

The first strategy consists in cultivating the microbial BC producer in the presence of alginate dissolved in the culture medium [32]. The BC–alginate composite is the result of interpenetrated chains of alginate with cellulose. Biophysical analyses of BC-Alg composite revealed the interactions of both polymers determined by Fourier Transform Infrared Spectroscopy (FTIR), concomitantly with a decreased crystallinity and increased surface and pore size.

A second technique is to deposit the polymer, Alg, over the BC film, followed by cross-linking with a divalent cation producing a binary composite [94]. This synthesis provides interactions between both polymers that are mainly physical, except in the contact surface between both biopolymers. The binary BC-Alg composite mostly keeps the main characteristics of its individual components, which means the crystalline structure of BC but with a reduced pore size, changeability of the BC surface, and high water absorption and holding compared with pure BC.

In a third reported approach, the BC film is crushed by a blender into small fibers, a mixture of bacterial cellulose nanofibers and microfibers (BC_f_), and is added to an alginate solution with agitation until total dispersion is observed, with the formation of a viscous slurry, and later cross-linked in the presence of calcium followed by freeze-drying [95]. The resulting BC_f_-Alg composite contains polydisperse pore sizes in the small range of 30–80 nm and the wide one from 1 to 340 µm, which are attributed to the inhomogeneous BC processing. In addition, there is a fourfold decrease in the tensile strength and an almost twofold one in the BC_f_-Alg compared with BC films. Moreover, the water uptake by BC_f_-Alg increases by about six times compared with BC films, concomitantly with poor structural stability when the BC_f_-Alg composites are immersed in watery solutions for one day [96]. The water solubility of the composite is a relevant parameter for the development of wound healing devices since they must be insoluble: the films should be able to absorb the body fluids from the injured area while keeping the product’s integrity.

The development of different strategies to make BC-Alg composites allowed the investigation of complex structures containing other polymers for wound healing or tissue repair by simultaneously improving more than one relevant property such as mechanical and biocidal properties, stability, water holding, etc. [96,97,98].

Besides the different strategies for making BC-Alg composites, all studies report their lack of toxicity, which makes them potential candidates to develop prototypes for many chronic and acute pathologies involved in wound healing and tissue repair [99].

### 4.2. BC–Collagen Composites

Collagen is the most abundant protein in mammals, found in the extracellular matrix, and comprises nearly 90% of the skin in humans. Collagen is composed of nonpolar glycine and proline or hydroxyproline in repetitive units arranged in repetitive triplets along a chain with more than 1000 amino acids [100]. Collagen is widely used in cosmetics, biomedical implants, and tissue engineering because of its low antigenicity, resistance to mechanical stress, and excellent cellular adhesion [100]. In addition, the composite of collagen (type I) was reported to reduce the levels of interleukins, reactive oxygen species (ROS), and proteolytic activity [101]. These characteristics make it a promising polymer to improve the mechanical and biological properties of BC.

Two common strategies of collagen incorporation into BC membranes were reported. Ex situ addition of collagen to BC membranes produces a composite with better tensile strength and Young’s modulus than only pure materials, but it also promotes the adhesion and proliferation of 3T3 fibroblast cells [102]. However, collagen can also be added in situ to the culture medium during bacterial cell growth. As a result, not only does the thickness of the BC membrane and its mechanical properties change but also its ability to load antimicrobial and antioxidant compounds to the membrane [103].

There are also other methods to generate hybrids of BC and collagen. BC pulp, generated by BC membrane trituration and co-precipitated with collagen, has shown excellent results in wound healing in in vivo studies. These hydrogels proved to enhance the orientation quantity and quality of collagen fibers synthesized by the damaged tissue; moreover, they also proved to maintain a humid environment in the wound, showing good adhesiveness, and accelerated wound healing [104].

Moreover, a composite made of BC–gelatin and denatured collagen improved cell proliferation and film adhesion to cells [105]. This pioneering work revealed the non-covalent interaction of primary amine groups of the gelatin with the hydroxyl groups of BC through FTIR spectroscopy, a decrease in the crystalline structure of BC that could increase the load of potentially therapeutic molecules for wound healing.

In another work, BC–gelatin was cross-linked with glutaraldehyde and revealed a uniform honeycomb surface with uniform pore size. This procedure with the bifunctional reagent covalently cross-links the hydroxyl groups of BC with the primary amine groups of the gelatin, forming covalent bonds through the aldehyde groups. The specific surface area of the composite was reduced by approximately five times, from 200 m^2^/g of BC to 40 m^2^/g of the BC–gelatin composite with an increase in the tensile strength and thermal stability [106]. Similarly, the development of BC–gelatin coacervate was performed with glutaraldehyde for the delivery of ampicillin. Moreover, ampicillin release from the BC–gelatin hydrogel displayed a non-Fickian release model [107]. In another study, BC–gelatin was cross-linked with glucose by heat. The authors claim that cross-linked BC–gelatin via the Maillard reaction displays good biocompatibility with Vero cells [108]. However, it is relevant to mention that the result of both cross-linking procedures (i.e., glutaraldehyde and glucose via the Maillard reaction) is considered toxic and sometimes difficult to eliminate by washing with aqueous solutions because of the hydrophobic properties of the residual compounds and the closed interpenetrated network of the composite.

### 4.3. BC–Chitosan Composites

Chitosan (Chi) is a low-cost, biodegradable, biocompatible polymer with antimicrobial properties [109]. Chi was approved to be used for wound dressing by the FDA (but it is still not considered GRAS) and is used in other countries for dietary purposes. Chi composites with BC are developed to enhance mechanical properties and antimicrobial activity. As with collagen, the addition of Chi to BC membranes can be performed in situ, ex situ, or by generating a hybrid hydrogel from the precipitation of a mixture of BC pulp and Chi. Regardless of the preparation method, the composites retain the antimicrobial capacity of Chi, have a more flexible structure than BC alone, and preserve the biodegradability and biocompatibility of both polymers [110,111,112]. In recent studies, BC membranes were immersed in Chi and Chi–ferulic acid solution to produce membranes with antimicrobial activity [112,113]. Biophysical studies using spectroscopic, SEM, gravimetric, and other techniques demonstrated that BC-Chi composites can hold more water compared with neat BC. The hybrid BC-Chi composite cross-linked with glutaraldehyde showed a high storage modulus in 20% BC, while the increase in BC decreases the storage modulus, which could be correlated with the decrease in the antimicrobial activity against *E. coli* and *S. aureus* because of low chitosan content in the film [112]. Moreover, in BC-Chi containing ferulic acid, the antimicrobial activity against *E. coli* and *S. aureus* was enhanced by the presence of the acid [113]. However, no toxicity analyses of BC-Chi composites were reported in either studies. The authors claimed that the BC membranes grafted with Chi could be used for wound healing and food packaging. An alternative application of chitosan for the development of hybrid BC systems employed chitosan oligosaccharides and Chi nanoparticles to produce films that had antimicrobial activity [114,115].

A comparative antimicrobial effect of hybrid BC composites containing Chi (MW ≈ 700–800 kDa) or Chi oligosaccharides (Chi_o_, MW ≈ 1.7 kDa) against *E. coli* and *S. aureus* showed high biocidal activity, but a 10% decrease in dead cells was observed in *E. coli* plates in the presence of Chi_o_. Alternatively, the BC-Chi_o_ and BC-Chi composite films showed approximately 92% and 58% antioxidant activity against ABTS, respectively. A similar antioxidant trend was observed in the presence of DPPH [114]. The high antioxidant activity of BC-Chi_o_ composites is relevant because the presence of radicals in wounds can be deleterious for the regeneration of damaged tissue.

In another interesting approach, water dispersions of polyvinyl alcohol (PVA) and BC_f_ suspension were mixed and dried. Water dispersions of Chi and Chi nanoparticles (Chi_n_) were added to the PVA-BC film. The analysis of the water solubility of PVA-BC_f_-Chi composites showed approximately a two and two and a half times increase in film solubility when the Chi concentrations incorporated in the composite increased from 0.5% to 1.0% and 2.5%, respectively. Alternatively, the water solubility of PVA-BC_f_-Chi_n_ was in the same range as PVA-BC with about a 20% difference. In the case of water vapor permeability (WVP), the addition of Chi to PVA-BCf reduced it between 3% and 10% when the cationic polymer increased from 0.5% to 2.5%, respectively. On the other hand, the addition of Chi_n_ diminished WVP by approximately 10% to 20% by increasing the nanoparticle concentration in the PVA-BC_f_ composite. The antimicrobial activity of both types of composites against *E. coli* and *S. aureus* displayed considerable differences. PVA-BC_f_-Chi showed low or no biocidal activity against *E. coli* and *S. aureus* except at the highest concentrations tested of 2.5% Chi. Meanwhile, all PVA-BC_f_ composites containing Chi_n_ displayed higher antibacterial activity against the two microorganisms than Chi films, also at the lowest concentration tested (i.e., 0.5%). These results are indicative of the antimicrobial effectivity of Chi nanoparticles, which is relevant because the toxicity of Chi and Chi_n_ increased with the concentration, polymer molecular weight, deacetylation degree, etc., as was reviewed recently with some controversial conclusions [116,117,118]. Moreover, Chi addition to BC films can improve the biocompatibility of membrane composites with 3T3 fibroblast and L929 fibroblast cell lines, and human keratinocyte cell adhesion has been demonstrated in this kind of composite [54]. All this considered, BC-Chi composites show promising results in the future of wound-healing treatments.

### 4.4. BC–Silk Sericin Composites

Silk sericin is a polar protein by-product of silk production that stimulates proliferative effects on fibroblasts and keratinocytes. Silk sericin ex situ addition produces membranes that not only have BC advantages (i.e., water absorption, water vapor permeation, biocompatibility, etc.) but also stimulate tissue regeneration. The follow-up of the healing of wounds in human patients treated with this type of composite did not show faster healing compared to the control waxed tulle dressing; however, it showed reduced inflammation markers (such as COX-2, IL-1β, and TNF-α), reduced referred pain, and scar improvement [119,120].

### 4.5. BC–Miscellaneous Composites

BC micro- and nanocomposites can also be made from BC membranes, fibers, many polymers, and inorganic substances. Among them, BC/silver composites are possibly the most studied in the field of wound healing to avoid microbial infections with potential septicemia. Silver has been used for many years in the topical treatment of acute or chronic wounds for its broad-spectrum antimicrobial properties without undesirable secondary toxic effects [121]. Strategies for forming BC/silver composites do not normally involve free silver ions but deposits of nanoparticles, microparticles, or other configurations of metallic silver or silver compounds, which implies that the release of silver ions from the membranes, if any, is very slow. As a consequence, while antimicrobial activity is retained, the chances of silver toxicity are low, and the durability of the membrane is high [33,37,122]. Recently, a new composite created by the in situ addition of silver nanowires was reported. This new composite showed high water retention and water vapor permeability like BC membranes, high stretchability, and accelerated in vivo wound healing in mice [123]. However, hybrids of BC with inorganic materials are not limited to silver. Composites with zinc oxide or titanium oxide have shown excellent antimicrobial activity against microorganisms that normally colonize wounds. Additionally, these composites were shown to accelerate re-epithelialization and burn healing in mouse models [27,124]. Composite preparations with these oxides can be achieved, of course, by ex situ methods. However, regenerated BC/zinc oxide films have been reported to have excellent biocompatibility, good antibacterial activity, cell adhesion capabilities, and improved mechanical properties [125].

However, composites are not restricted to BC membranes and fibers. Cellulose nanocrystals have been shown to improve the mechanical properties of chitosan films; the anionic groups of nanocrystals interact with the amino groups of chitosan and increase the continuity between the polysaccharide chains, which improves the tensile strength and elastic modulus of the films [126]. Additionally, bacterial cellulose nanocrystals can act as amphiphilic material, ensuring a sufficiently stable interaction between hydrophilic materials and other hydrophobic materials or hydrophobic cargos. This concept has been put to the test in the preparation of emulsions containing alginate, BC nanocrystals, and a highly hydrophobic drug, alfacalcidol (i.e., a vitamin D precursor) that showed low cytotoxicity and sustained release capabilities [127].

In the foregoing, we reviewed some composites with bacterial cellulose that present its application in the healing of wounds. For example, there is an extensive study on the development of BC composites and graphene nanotubes. These composites have improved electrical, mechanical, and thermal properties, with possible applications in biosensors and drug delivery. However, the tendency of carbon nanotubes to aggregate in physiological media raises some concerns, and application in biomedical devices, although very promising, will require an exhaustive study of their biocompatibility [128]. Additionally, composites have been reported with several other substances, such as poly(lactic acid) [129], polyurethane [130], calcium phosphate [131], and even composites of cellulose with different treatments [70], among many others [63]. Taken together, they paint a picture in which the preparation of composites is not only the tool to overcome the disadvantages that BC could have but also introduces new properties for application in tissue regeneration in general and specifically in wound healing. Table 1 summarizes the composites reviewed in this section and their properties.

## 5. BC for Wound Dressing

In the case of dressings to resolve the clinical scenario of chronic wounds, bacterial cellulose, in comparison with traditional dressings, presents interesting characteristics in the treatment of skin lesions, such as immediate pain relief, the maintenance of local humidity that prevents the formation of crusts, its action as a physical barrier of protection that reduces the rate of external contamination, and the adsorption of exudates during the inflammatory phase, among other characteristics that accelerate the healing process and shorten the time of treatment of the lesion [131,132,133]. In addition, BC does not have other components such as lignin, pectin, or hemicellulose, present in plant cellulose, which facilitates its use for biomedical purposes.

In the study by Silva et al. [134], a randomized controlled clinical intervention study of venous ulcers of the lower limbs using bacterial cellulose in the form of a membrane, gel, and multiperforated film was performed. The experimental group (EG), with 20 patients, was treated with BC-based dressings, and the control group (CG), with 19 patients, was treated with dressings made of a cellulose acetate mesh impregnated with essential fatty acids (RAYON^®^). The BC-based dressings were able to reduce the initial wound area significantly, similar to the commercial dressing (RAYON^®^), which required fewer interventions and manipulations of the injured area, reducing the risk of contamination. Moreover, a decrease in local pain was reported, and greater ease in using the CB-based dressings brought greater autonomy and well-being to patients.

In the study by Wahid et al. [135], bacterial cellulose was functionalized with polydopamine (PDA) followed by the incorporation of ε-polylysine (ε-PL), named BCP@ε-PL, for the development of a possible dressing for infected wounds. Through in vitro tests, it was possible to observe strong antibacterial properties in addition to the hemo- and cytocompatibility of BCP@ε-PL. In in vivo tests, it was also possible to observe antibacterial properties and a complete healing of the wounds treated with the functionalized films in comparison with the isolated BC and the control group which remained open in the same period. These results revealed that the functionalized membranes have great potential as a dressing material for infected wounds in future clinical applications.

In the study by Asanarong et al. [136], papain, an agent with known antibacterial properties isolated from papaya latex, was incorporated into the CB (BE) and the composite based on CB and glutaraldehyde was obtained (BEG). The cellulose films and the composite containing papain (BE and BEG, respectively) showed the ability to inhibit in vitro the growth of Gram-positive and Gram-negative bacteria. However, based on agar diffusion tests, BEG composites had twice the antibacterial capacity compared with that of BE. BC films containing papain showed promising results as bioactive dressings in the treatment of infected wounds and acne.

Another composite, now using bacterial cellulose and polycaprolactone (BCP), has been researched as a possible dressing with antibacterial properties. In the study by Das et al. [137], a flexible composite (BCP) was obtained that was functionalized with antibiotics gentamicin (GEN) and streptomycin (SM). The materials exhibited antimicrobial properties against bacteria *E. coli* and *S. aureus* and were biocompatible and non-toxic to BHK-21 cells even after 72 h. In addition, the drug release profiles indicated a uniformly distributed BCP scaffold, which could be used as a wound dressing material.

## 6. Biocide Formulations of BC

Biocide Bacterial Cellulose

Dressings with an antimicrobial load have attracted great interest in the field of wound healing due to site-specific distribution, reduced adverse effects, increased concentration at the target site, low incidence of resistance, and the use of agents not suitable for oral and systemic therapy [138,139,140,141]. Among them, BC membranes are well recognized and effective scaffolds for wound healing and have recently been reviewed in high detail [33,122,123]. However, cellulose membranes do not have any protective or biocide activity and can only provide a physical barrier to potential invading organisms, limiting their use in infected wounds [31,32]. Additionally, the large pore size of BC membranes, approximately 31 nm, does not allow for the retention of small molecules [32]. Because most biocides are low-molecular-weight chemical structures (i.e., MW lower than 1000 Da), their average diameter is lower than approximately 2 nm. Considering these characteristics, low-molecular-weight biocides without any interaction with the BC membrane can freely diffuse out of the membrane through a dominant release mechanism of Fickian diffusion. Strategies to produce BC dressing scaffolds with biocide action and controlled drug release must involve some biophysical and/or biochemical modifications of the membrane. BC membrane modifications can generate multifunctional dressing structures that help in several aspects, such as reducing local pain, shortening the inflammatory phase, accelerating healing, and the fast healing of damaged tissue.

The development of BC displaying biological activities includes the chemical modification of the biocides to provide interaction within the cellulose chain of BC or by the addition of macromolecules with or without the biological agents.

In general, the inclusion of small bioactive molecules in the pure BC films can be effective in a limited range of time when there exist chemical interactions between the cellulose chains and the supplemented molecule. In such cases, a bulk release of a bioactive molecule can be seen because the release is only mediated by free diffusion. Additionally, the concentration of the bioactive molecule is limited by the width of the therapeutic window and toxic effects can be observed. In particular, this technique limited the use of the BC scaffold at the time because the cellulose films lost their biological activity very quickly. Moreover, in the case of modifying biocide molecules to obtain interactions with the BC, regulatory issues with national and international agencies (i.e., the FDA, European Medicines Agency (EMA), etc.) can limit the clinical uses because of the potential presence of toxic residues after the covalent derivatization.

Modification of BC scaffolds with polymers for controlled release is mainly produced by two main strategies: in situ or ex situ. In the former, the modifier molecule must be added to the culture medium during bacterial growth. Additionally, some minimal requirements of the exogenous molecule must be considered for addition to the bacterial culture, such as lack of toxicity for the growing microorganism, compatibility with the media and their components, stability under environmental conditions, and, importantly, solubility in the medium under the defined operational conditions [142]. The main drawback of this strategy is the purification step of the BC composite at the end of bacterial growth. In general, BC composites are purified to eradicate microbial cell debris and potential toxic molecules, which requires the use of strong bases/acids. Consequently, the additive molecule can be partially degraded and/or altered in its physicochemical properties. The main advantage of this procedure is the high interpenetration of the BC fibrils with the exogenous molecule, generally a polymer, during the formation of the BC composite. The incorporation of the exogenous molecule, generally a macromolecule, changes the structure of the nascent BC fibrils, modifying the pore size and volume, crystallinity, and tensile strength among other biochemical properties such as its free functional groups, mucoadhesiveness, drug–matrix components interactions, and the kinetics of molecular controlled release [31,32,35].

The second strategy or ex situ BC modification is very simple and does not include the limitations of the in situ requirements. After BC production and purification, the solution containing the modifier molecule, generally a polymer, can be placed in close contact with the membrane. The exogenous macromolecule interacts mainly with the BC surface and can diffuse through the membrane pores. The filler molecule covers the holes of the membrane pores but mostly the BC surface. The changes in the physicochemical properties of the BC composite depend on the degree of filler integration within the membrane [23,24,25,35,46]. However, the interaction between the BC membrane and the filler is weaker compared with the in situ procedure if no covalent chemical modification is performed. Since the main mechanism of the molecular deposition of the exogenous molecule over the surface of the BC membrane follows Fick’s laws, some limitations, such as in its molecular weight and chemical interaction between the BC membrane and the exogenous molecule can be detected. Therefore, the penetration of the molecular filler into BC depends on the concentration, molecular weight, functional free groups to interact within the cellulose matrix, and viscosity of the filler solution but also on the experimental conditions (e.g., temperature, pH, etc.). Moreover, the exogenous molecule must be compatible with the BC membrane and any other molecules to be loaded in the composite.

Incorporation of bioactive molecules (i.e., antibiotics, growth factors, proteins, enzymes, etc.) into the modified BCs can follow two main strategies. In one case, the bioactive molecule must be added after the BC composite is formed. This is the case of biocides in general, such as anti-fungal, anti-bacterial, and anti-parasitic ones, which are molecules of low molecular weight. The molecular interactions of the bioactive molecule with all of the components of the BC composite will determine the main therapeutic properties of the scaffold. It is important to prevent incompatibilities, such as partial or total inactivation and/or degradation of the bioactive compound/s. In particular, the main process of bioactive molecule incorporation into the BC ex situ modified scaffolds will be by diffusion, followed by surface adsorption. Moreover, the physicochemical properties of the bioactive molecule (i.e., molecular weight, chemical composition, available functional groups, and folding in the case of proteins and enzymes) will determine the final location of the molecule in the BC composite. However, the main drawback of adsorbed molecules on BC composites is the exposure of bioactive molecules to the environmental conditions when there are mainly located on the BC composite surface.

The reactivity of bioactive molecules can be affected by diverse physiological situations (e.g., shear rate, pH, ionic strength, and/or temperature changes) and by the effects of shear rate on molecular structure and folding. Additionally, if the main interaction of the bioactive molecule with the BC composite is through an adsorption mechanism, a bulk release could be expected since the adsorptive mechanism involves weak molecular interaction forces and depends on environmental conditions. In some cases, to solve this problem, a cross-linker (e.g., EDC, glutaraldehyde, genipin, etc.) will be added to form covalent bonds between the molecule and the matrix.

Another strategy for the incorporation of a bioactive molecule/s into the BC involves the previous mixing of the bioactive agent/s with the exogenous molecule, generally a polymer, followed by loading the mixture to the BC membrane surface. The activity, stability, and kinetic release of the bioactive molecules will be mainly dependent on the interaction within the molecular filler and the release mechanisms of the mixture components into the BC membrane.

Many cases of BC composites developed for the delivery of biocides were recently reviewed [100,108,109,110,111,112,113,114,115]. A few additional examples of BC-modified scaffolds developed for wound healing and using different strategies are detailed below.

A modified montmorillonite–bacterial cellulose nanocomposite, to be used as a new replacement for burned skin and tissue regeneration, was reported [24]. The syntheses of modified montmorillonites (MMTs), a typical type of clay, were carried out by the ion-exchange reaction method using Cu, Na, and Ca. Then, the nanocomposites were prepared with BC. It was concluded that the modified nanocomposites showed good antimicrobial activity against pathogens associated with burns and improved wound healing and tissue regeneration compared with BC and MMT-BC nanocomposites in animal models. In another study, first MMT ions were exchanged with Ag^+^ to provide the clay with biocide activity in the nanocomposite. Later, the MMT-Ag was added to the BC scaffold and tested against *Staphylococcus aureus* (Gram-positive bacterium) and *Pseudomonas aeruginosa* (Gram-negative bacterium), showing high antimicrobial activity. BC nanocomposites containing MMT-Ag, in the range of 1.0 to 25.0%, were biocompatible with L929 fibroblasts [33]. Furthermore, BC-MMT can be applied to mobile parts of the body, such as the knee and elbow, due to the flexibility of BC. The clay nanocomposites will also increase the scaffold stability and ensure better patient compliance as needed, require fewer changes, and a single application will reduce pain after wounds due to their soothing properties and painless removal.

Another interesting strategy was the preparation of antimicrobial BC–silver nanoparticle composite membranes using triethanolamine as a reducing and complexing agent [143,144]. It was observed that the composite membranes presented a strong antimicrobial activity against *Staphylococcus aureus*, *Pseudomonas aeruginosa,* and *Escherichia coli* (Gram-negative bacteria), which are the main microorganisms in normal skin but often found in contaminated wounds. Similarly, the synthesis of silver nanoparticles, performed in a solution containing hydroxypropyl-β-cyclodextrin (HßCD), and curcumin as a natural reducing agent, was reported [139]. Later, the nanoparticles and HßCD complex were then loaded in bacterial cellulose hydrogel with moist wound healing properties, resulting in a new dressing platform. The dressings exhibited antimicrobial activity against *Staphylococcus aureus*, *Pseudomonas aeruginosa,* and *Candida auris*, three common pathogenic microorganisms that infect wounds. Furthermore, they showed high cytocompatibility.

Another strategy to synthesize composites using silver as a biocide was to take advantage of the asymmetric property of BC films. The surface of BC at the interphase liquid–air is flat; meanwhile, the BC surface inside the liquid culture contains the cellulose pending chains of the fibers, taking advantage of the cellulose pending chains that can be used as a seed for the precipitation of water-insoluble salts. Silver phosphate is very insoluble in aqueous media, with a very low constant of solubility product (i.e., K_ps_ = 1.8 × 10^−18^). The addition of silver ions to BC suspended in phosphate buffer showed asymmetric precipitation in the form of microparticles on the pending chain of cellulose fibers. The BC–silver phosphate membranes showed high antimicrobial activity against *E. coli* and *S. aureus*, making it a potential excellent scaffold for wound healing [78].

In another relevant study, bacterial cellulose membranes impregnated with silver sulfadiazine showed high antibacterial activity against *P. aeruginosa*, *E. coli,* and *S. aureus*, and it was proved to be biocompatible with epidermal cells and can be used as an antibacterial dressing for the treatment of skin wounds [143].

Another interesting study proposed the use of BC and diethyldithiocarbamate (DETC) for the treatment of cutaneous leishmaniasis (CL) wounds caused by *Leishmaniasis braziliensis* [145]. After loading BC with different amounts of DETC, the nanocomposite was dried to enhance the adsorption of the drug on BC membranes. Infected wounds in the ear region of mice were tested with the BC-DETC scaffolds. The lesion size exposed to BC-DETC was significantly reduced after 2 weeks. Meanwhile, the same effect was not observed in the control group with only BC. In addition, a reduction in the parasite load and inflammatory response at the infection site were seen. This material is promising for the treatment of cutaneous leishmaniasis in humans.

A relevant study of BC used for the transdermal release of ibuprofen modified with amino acid alkyl esters using porcine skin was reported [22]. By modifying ibuprofen, it was possible to control the permeability rate of the drug in the skin, indicating the great potential of BC for use in the transdermic release system.

## 7. Final Conclusions

An extensive number of academic publications recently showed interest in BC for many industrial applications, particularly in the biomedicine area, and were recently reviewed [33,74,76,77,80,82,122,132,135,142,146,147,148,149,150]. BC can be produced from many sources, including wastes of different origins to obtain many neat cellulose biocompatible structures [48,108]. Among the advantages of BC is its highly crystalline structure compared with plant cellulose, as well as the many alternatives to render it, from simple films to very complex structures, by processing the films using different molecules from antibiotics to polymers, inorganic complexes, and salts. Moreover, BC polymer chains can be chemically and enzymatically modified, giving a myriad of complex and active biomedical structures.

Several new bacterial-cellulose-based dressings were reported to have antibacterial properties [135,136,137], with increased healing and re-epithelialization capacity [138,139,140], demonstrating the advantage of using the biopolymer in the production of new materials for biomedical use. The relevance of different BC structures to wound healing reflects the variety of technological alternatives for the treatment of wound healing and tissue repair, many of which are prototypes, and some are now on the market [147,151]. Since wound healing and tissue repair are of high importance and social interest, BC seems to be an excellent starting material for the development of devices that can be extensively used in biomedicine.

## Figures and Tables

**Table 1:** Bacterial cellulose composites are included in this review.

Compound	Technique	Properties	References
Alginate	In situ	Enhanced surface and pore size	[32]
	Ex situ	Higher WHC	[90]
	Blend and cross-linking (calcium)	Improved tensile strength and WHC; decreased water stability	[91]
	BC nanocrystals in alginate film	Increased drug loading capacity	[124]
Collagen	Ex situ	Enhanced tensile strength and Young’s modulus; promotes fibroblast adhesion and proliferation	[98]
	In situ	Enhanced mechanical properties; increased drug load capacity	[99]
	Blend	In vivo accelerated wound healing; enhanced wound collagen orientation	[100]
Gelatin	Ex situ	Enhanced Young’s modulus; decreases tensile strength and WHC. Improved fibroblast proliferation	[101]
	Blend and cross-linking (glutaraldehyde)	Increased thermal and mechanical stability; increased drug loading capacity	[102]
	Blend and cross-linking (glutaraldehyde)	Uniform pore distribution; sustained drug release	[103]
	Blend and cross-linking (glucose)	Improved mechanical and thermal properties	[104]
Chitosan	Blend and cross-linking (glutaraldehyde)	Increased thermal stability; antimicrobial activity	[109]
	Ex situ	Improved mechanical properties; enhanced swelling behavior; antimicrobial activity	[110,111]
	Blend + PVA	Improved mechanical properties, antimicrobial activity, and UV opacity	[112]
	BC nanocrystals in chitosan film	Improved tensile strength and elastic modulus	[123]
Sericin	Ex situ	Reduced inflammation markers; reduced referred pain in clinical trials; better scar improvement	[116,117]
Silver compounds and/or structures	Diverse strategies	Antimicrobial activity	[54,119,120]
Silver nanowires	In situ	In vivo accelerated wound healing	[121]
Zinc oxide, Titanium oxide	Ex situ	Accelerated burn healing in mouse models	[33,122]
	Regenerated blend	Improved mechanical properties and antibacterial activity; enhanced cell adhesion	[71]

## Abbreviations

Alg	Alginate
BC	Bacterial Cellulose
BC_f_	Bacterial Cellulose Nanofibers and Microfibers
BNC	Bacterial Nanocellulose
BSA	Bovine Serum Albumin
CAGR	Compound Annual Growth Rate
Chi	Chitosan
Chi_o_	Chitosan Oligosaccharides
Chi_n_	Chitosan Nanoparticles
CL	Cutaneous Leishmaniasis
CNCs	Cellulose Nanocrystals
CNFs	Cellulose Nanofibers
CTs	Bacterial Cells
DETC	Diethyldithiocarbamate
DMAc -N	N-dimethylacetamide
EMA	European Medicines Agency
FDA	Food and Drug Administration
FTIR	Fourier Transform Infrared Spectroscopy
GRAS	Generally Recognized as Safe
MTT	Montmorillonite
NMMO	N-methylmorpholine-N-oxide monohydrate
PC	Plant Cellulose
PEG	Polyethylene Glycol
PLGA	Poly (L-lactide-co-glycolide)
PU	Pressure Ulcers
PVA	Polyninyl Alcohol
RBC	Regenerated Bacterial Cellulose
ROS	Reative Oxigen Species
RPC	Regenerated Plant Cellulose
WHC	Water-Holding Capacity
WHO	World Health Organization
WRR	Water Retention Rate
WVTR	Water Vapor Transmission Rate
